# Cooperative interactions in the *West Nile virus* mutant swarm

**DOI:** 10.1186/1471-2148-12-58

**Published:** 2012-05-22

**Authors:** Alexander T Ciota, Dylan J Ehrbar, Greta A Van Slyke, Graham G Willsey, Laura D Kramer

**Affiliations:** 1 New York State Department of Health, Wadsworth Center, Slingerlands, NY, USA; 2 Department of Biological Sciences, State University of New York, Albany, NY, USA; 3 School of Public Health, State University of New York at Albany, Albany, NY, USA

## Abstract

**Background:**

RNA viruses including arthropod-borne viruses (arboviruses) exist as highly genetically diverse mutant swarms within individual hosts. A more complete understanding of the phenotypic correlates of these diverse swarms is needed in order to equate RNA swarm breadth and composition to specific adaptive and evolutionary outcomes.

**Results:**

Here, we determined clonal fitness landscapes of mosquito cell-adapted *West Nile virus* (WNV) and assessed how altering the capacity for interactions among variants affects mutant swarm dynamics and swarm fitness. Our results demonstrate that although there is significant mutational robustness in the WNV swarm, genetic diversity also corresponds to substantial phenotypic diversity in terms of relative fitness *in vitro*. In addition, our data demonstrate that increasing levels of co-infection can lead to widespread strain complementation, which acts to maintain high levels of phenotypic and genetic diversity and potentially slow selection for individual variants. Lastly, we show that cooperative interactions may lead to swarm fitness levels which exceed the relative fitness levels of any individual genotype.

**Conclusions:**

These studies demonstrate the profound effects variant interactions can have on arbovirus evolution and adaptation, and provide a baseline by which to study the impact of this phenomenon in natural systems.

## Background

The rapid replication rates, inherently high error rates, and large population sizes of RNA viruses often result in highly genetically diverse mutant swarms within individual hosts. A number of studies with arthropod-borne viruses (arboviruses) have demonstrated that the precise composition and breadth of these swarms may be directly responsible for alterations in viral fitness, pathogenesis, and host breadth [1]. Taken together, these studies demonstrate that a more comprehensive understanding of mutant swarm dynamics is required for adequate characterization of arbovirus evolution and adaptation. If the maintenance of highly diverse swarms equates to increased phenotypic flexibility, then swarm size could be particularly important for arboviruses given their requirement for replication in disparate hosts and tissues. Not surprisingly, many studies have identified significant intrahost diversity of arboviruses both in laboratory-derived and naturally occurring strains [2-5], yet the phenotypic diversity that correlates to these mutant genotypes has not been adequately characterized. Quasispecies theory, although often invoked as a synonym for genetic diversity, generally argues against the maintenance of substantial phenotypic diversity, as the inevitable coupling of mutational neighbors should ultimately favor selection for canalized populations which exhibit phenotypic stability in the face of mutational change [6-8]. As such, a robust viral swarm would theoretically lack substantial phenotypic diversity and therefore tend to negate the adaptive advantage one would normally associate with genetic diversity. A virus could overcome this need for robustness by readily utilizing cooperative interactions via strain complementation in co-infected cells.

Historically, discussion of complementation among viral proteins has focused primarily on defective interfering particles. The capacity for these incomplete genomes to hijack proteins from viable viral particles for replication has been documented extensively in cell culture systems, particularly during persistent infections [9]. In addition, previous studies with *Dengue virus* have demonstrated the maintenance of nonviable deletion mutants in both *Aedes aegypti* mosquitoes and acutely infected humans [10,11], and recent work with *West Nile virus* (WNV; *Flaviviridae*: *Flavivirus*) infected *Culex quinquefasciatus* also suggests that complementation may act to maintain deletion mutants in this system [12]. These studies together suggest that arboviruses in natural systems have the capacity to utilize complementation in co-infecting cells. Although these examples would be defined as ‘cheating’ from an evolutionary perspective, such interactions could also readily occur among variants with more intermediate fitness levels and in turn provide a net benefit to an arboviral swarm that encounters diverse environments in a single transmission cycle. Although previous studies provide evidence that viable genomes of *Vesicular stomatitis virus* (VSV) can utilize complementation [13], consideration of the importance of this phenomenon in the evolution of other medically important arboviruses is lacking. Such interactions could permit arboviruses to maintain genotypes which are host specialized, therefore diminishing the potential for compromises in host adaptation as a result of host cycling. Indeed, a number of studies have now clearly demonstrated that although host-specific adaptation may at times result in a fitness cost, adaptive trade-offs in individual hosts are not inevitable for arboviruses [14-21].

A previous experimental evolution study conducted in our laboratory generated a strain of WNV by sequential *in vitro* passages which was both highly adapted to mosquito cells and highly genetically diverse (WNV CP40). The breadth of the WNV CP40 mutant swarm was shown to be directly correlated with virus adaptation, and reverse genetics studies demonstrated that consensus mutations were not solely responsible for the adaptive phenotype, indicating the importance of mutant swarm variants in host adaptation and overall viral fitness [19,22]. Here, we sought to evaluate the level of robustness in this strain by quantifying the phenotypic diversity and relative fitness values of biologic clones derived from this WNV population. This strain provided a unique opportunity to characterize non-consensus variants of an adapted and genetically diverse arbovirus which had sufficient time to reach an equilibrium state. In addition, we tested the hypothesis that strain complementation plays a significant role in the maintenance of both genetic and phenotypic diversity in the WNV mutant swarm. Our results represent a significant advancement in the understanding of the relationship between swarm composition and viral fitness and the potential role of cooperative interactions in shaping arboviral mutant swarms.

## Results

### Distribution of relative fitness values of clonal populations of mosquito cell-adapted WNV

**Figure 1:**
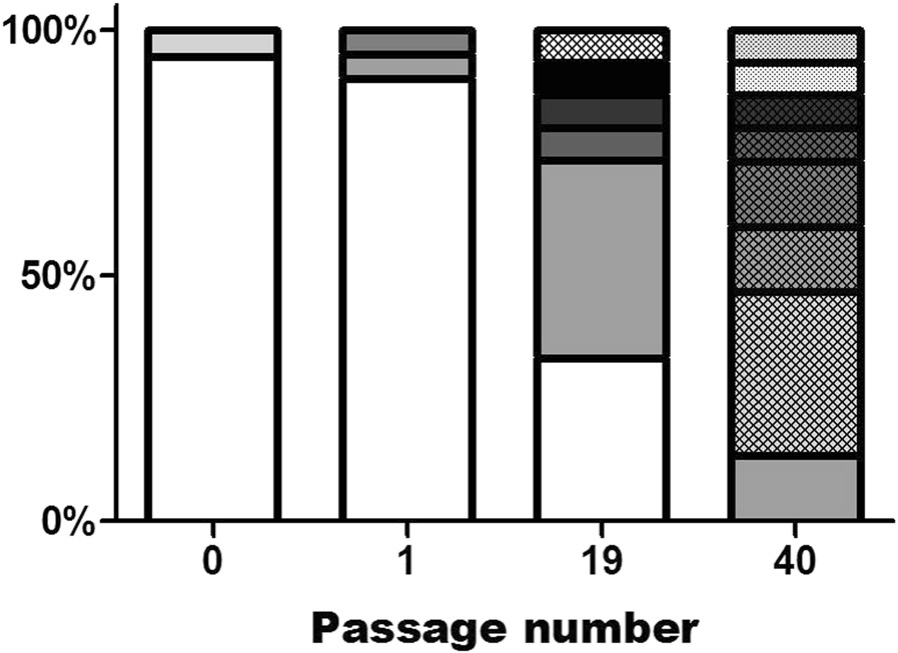
**Haplotype accumulation of*West Nile virus*during passage in mosquito cell culture.** Unique shades and/or patterns represent unique haplotypes identified by high-fidelity molecular cloning and sequencing of 15-20 clones of nt 1311-3248 following passage on C6/36 mosquito cells (some data modified from [22]).

**Figure 2:**
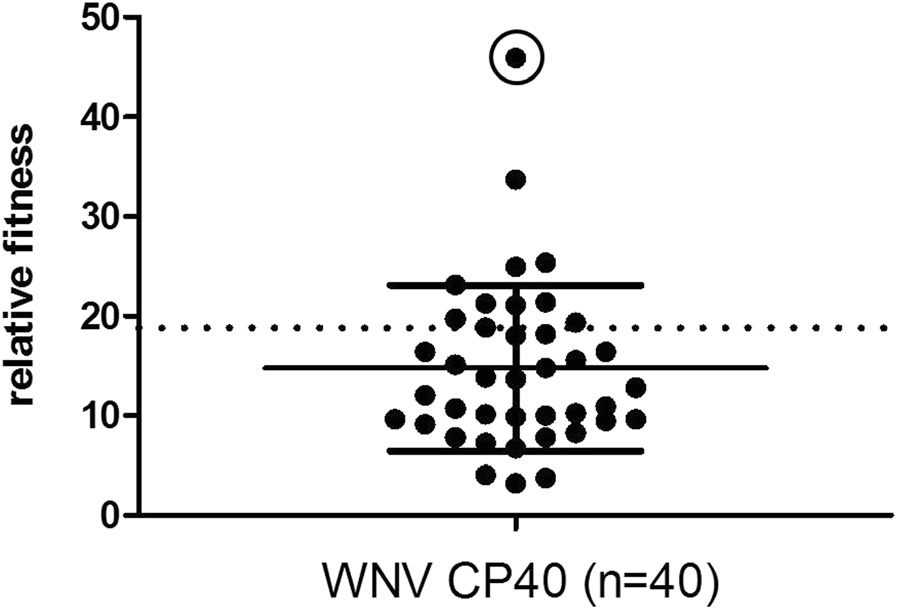
**Distribution of relative fitness values among 40 biological clones of WNV following passage on mosquito cell culture (WNV CP40).** Relative fitness refers to the proportion of individual WNV CP40 clones relative to the control virus (WNV MARM) following 72 h of competition on mosquito cell culture. The solid line represents the mean +/- standard deviation and the dotted line represents the fitness of the combined population. The circled data point represents the highest fitness clone isolated, WNV CP40-12.

Previous studies have demonstrated that the accumulation of minority variants in the WNV mutant swarm is coupled with passage and adaptation to mosquito cell culture ([22]; Figure 1). In order to assess the extent to which this genetic diversity corresponds to phenotypic diversity, 40 biologic clones of WNV CP40 were isolated and the fitness of individual variants was quantified using previously established methods. All variants tested out-competed the WNV monoclonal antibody resistant strain (MARM) strain, which was previously shown to have a fitness value equivalent to the parental strain from which WNV CP40 was derived [19]. Relative fitness values ranged from 3.2 to 45.9 (WNV CP40-12), with a mean value of 14.3 and a median value of 13.2 (Figure 2). Despite this phenotypic variation, 50% of variants had relative fitness values between 10 and 20. Following mixing at equal proportions, the relative fitness value of the population was 18.6, which was higher than both the mean and median values and 72.5% of the individual variants.

### WNV infectivity in mosquito cells

In order to assess if the level of co-infection was sufficient to allow the potential for interaction among viral genomes and/or proteins, qRT-PCR and flow cytometry were used to determine the mean number of viral particles per cell. Results demonstrated that following a 1-h infection of mosquito cells with WNV CP40 at a multiplicity of infection (MOI) of 10, approximately 7.4 log_10_ WNV RNA particles entered the cells, representing 29.2% of the virus inoculum (8.0 log_10_ particles/7.3 log_10_ pfu). Flow cytometry identified 68.2% of the cells in the monolayer as WNV positive, which is equivalent to approximately 6.1 log_10_ mosquito cells. Taken together, these results demonstrate an approximate mean particle to cell ratio of 14.3:1, signifying widespread co-infection of individual mosquito cells at high MOIs (Table 1).

**Table 1:** Infectivity and co-infection of WNV CP40 in mosquito cells

WNV particles-inoculum	Remaining WNV particles -1 h.	Total infectious WNV particles (%)	Cells infected -12 h (%)	Mean particle/ cell ratio
1.0 X 10^8^	8.0 X 10^7^	2.0 X 10^7^ (29.2)	1.4 X 10^6^ (68.2)	14.3

### Relationship between levels of co-infection and relative fitness

**Figure 3:**
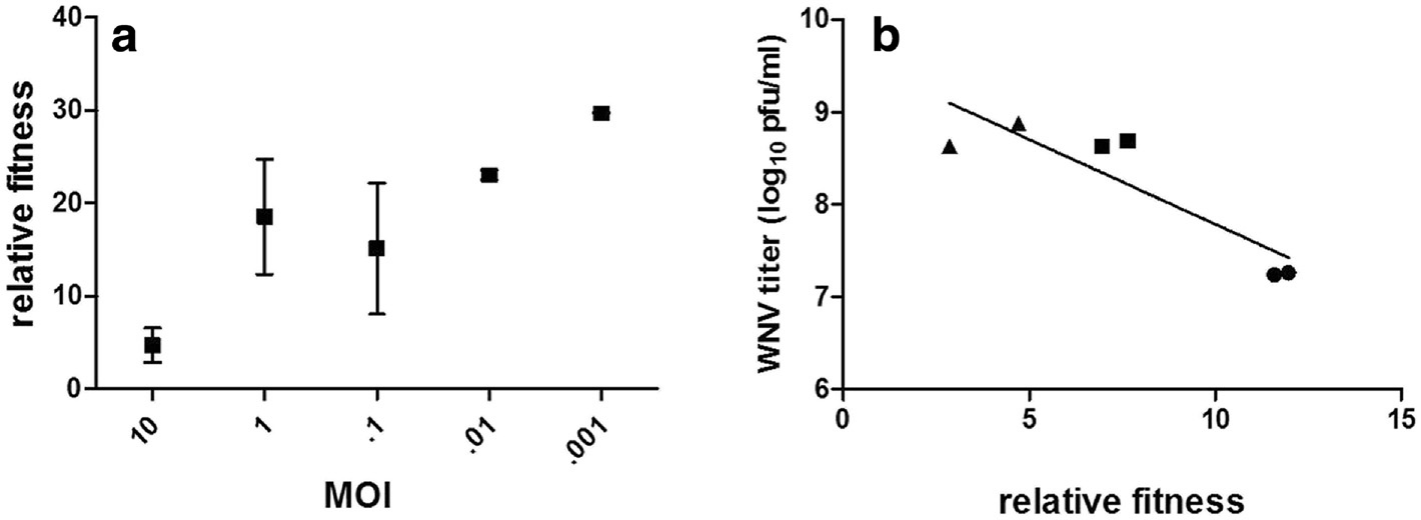
**Relationship between levels of co-infection and relative fitness of WNV CP40-12 on mosquito cell culture.** a. Individual points represent relative fitness values +/- standard error of duplicate competition assays carried out for 72 h at indicated MOIs. A significant negative correlation between MOI and relative fitness was measured (linear regression analysis, r^2^ = 0.68, p = 0.0035). b. Relationship between WNV titer and relative fitness during competition following infection at an MOI of 10. Individual points represent duplicate measurements of output virus following 24 (circles), 48(squares), or 72 (triangles) hours of competition. Results demonstrate a negative correlation between WNV titer and relative fitness (Pearson correlation, r = -0.88, p = 0.0195).

*In vitro* competition assays at MOIs 0.01, 0.1, 1.0, and 10 were performed using two distinct fitness variants (WNV MARM and WNV CP40-12) to assess the relationship between co-infection and the capacity of high fitness WNV variants to out-compete lower fitness variants. In a population of variants which are independently competing, the highest fitness variant, in this case CP40-12 (circled value, Figure 2), should experience a swift selective sweep. The goal of these studies was therefore to assess the role of complementation in inhibiting this selective sweep. The results demonstrate a significant negative correlation between MOI and relative fitness values, indicating that the capacity for the lower fitness variant to be retained in the population is increased with increased co-infection (Figure 3a; linear regression analysis, r^2^ = 0.68, F = 16.68, p = 0.0035). Conversely, this indicates that a strain (CP40-12) which in the absence of co-infection achieves titers over 30 times higher than its lower fitness competitor (WNV MARM) would be subject to an approximately 6-fold decrease in its capacity to out-compete under these conditions when levels of co-infection are high (MOI 10 relative fitness < 5, Figure 3a). It should be noted that the output viral titers for these competition assays were similar at 72 h, suggesting that the absolute fitness of the combined infection is likely comparable and that changes only reflect different proportions of the two strains. Measuring relative fitness at 72 h post infection allowed such estimations of the overall replicative ability of the ‘population’, yet it is possible that differences in levels of replication could also be used to explain different relative fitness values (i.e. high MOI infections more quickly become saturated and may be less capable of out-competing). Thus, to confirm the role of co-infection in MOI-dependent fitness values, relative fitness was also quantified at intermediate time points for the MOI 10 competition assay. Results indicate an inverse relationship between viral titers and relative fitness values, demonstrating replication and secondary infection cannot explain differences in relative fitness and providing further support for the negative correlation between co-infection and the capacity for high fitness variants to out-compete (Figure 3b; Pearson correlation, r = -0.88, r^2^ = 0.78, p = 0.0195).

### Relationship between levels of co-infection and genetic diversity

**Figure 4:**
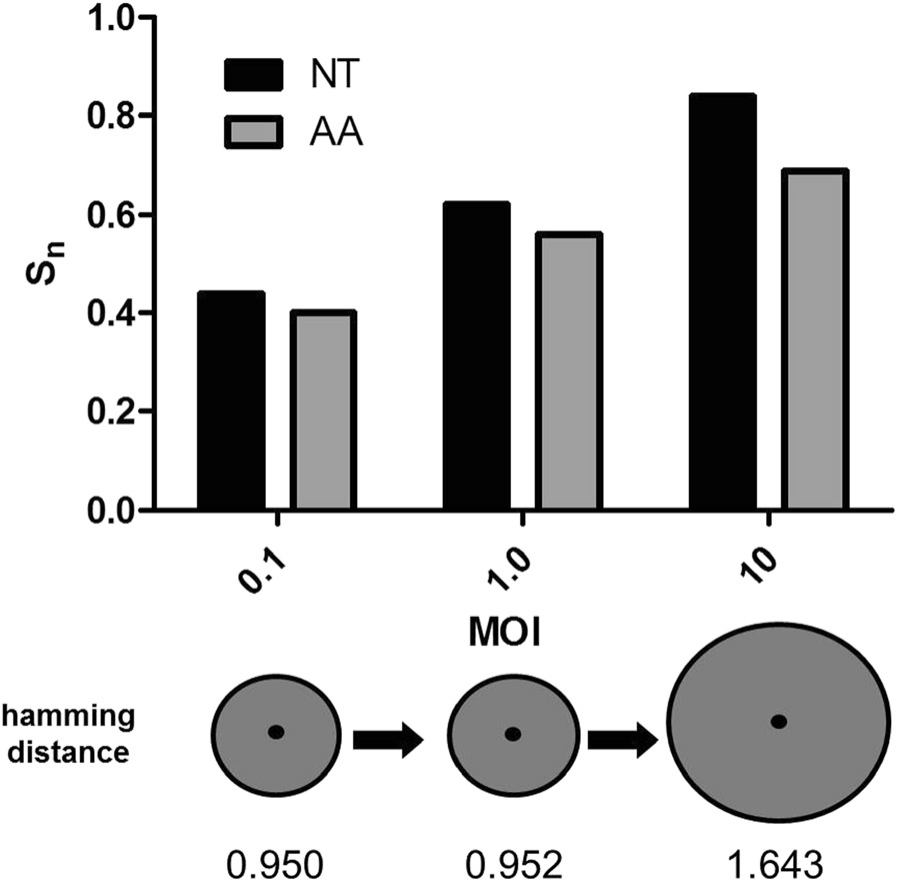
**Relationship between levels of co-infection and genetic diversity of WNV.** Bars depict normalized Shannon entropy (S_n_) on both the nucleotide (nt) and amino acid (aa) levels and circles depict relative differences in mean hamming distance for populations following a single passage of WNV CP40 at indicated MOIs. All measures of genetic diversity are for nt 1311-3248 and 17-22 clones were sequenced and analyzed for each measure. A significant positive correlation between co-infection (MOI) and S_n_ for both nt and aa was measured (linear regression analyses, r^2^ = 0.99, p < 0.05).

To directly assess the role of co-infection in maintaining genetic diversity, mutant swarm breadth was quantified after 24 h growth following infection at MOIs of 0.1, 1, and 10. Performing these experiments at 24 h eliminated concerns about secondary infection significantly altering the outcome. Analyses of approximately 20% of the genome following cloning and sequencing of output populations clearly demonstrate a significant positive correlation between MOI and multiple measures of genetic diversity (Figure 4). Specifically, comparisons of normalized Shannon entropy on both the nucleotide and amino acid level significantly increased with increasing levels of co-infection (linear regression analysis, r^2^ = 0.99 for both measures, p < 0.05). In addition, mean hamming distance of the MOI 10 mutant swarm (1.64) was significantly higher than both the MOI 1.0 (0.95; *t*-test, t = 2.2, df = 34, p = 0.037) and MOI 0.01 (0.95; t = 2.1, df = 34, p = 0.043) swarms, indicating that not only were more haplotypes present, but that individual variants were on average more different from the consensus sequence when co-infection was permitted. If the region analyzed is a reliable representation of the level of diversity genome-wide, the mean hamming distance for the full genome of individual variants is likely greater than 10 (WNV full genome ~ 11 kb). Multiple sequence comparisons of variants from the MOI 10 group performed with the Recombination Detection Program v.4.13 [23] did not indicate that recombination contributed to the increased levels of genetic diversity observed with increased co-infection.

### Relationships between co-infection and fitness distributions

**Figure 5:**
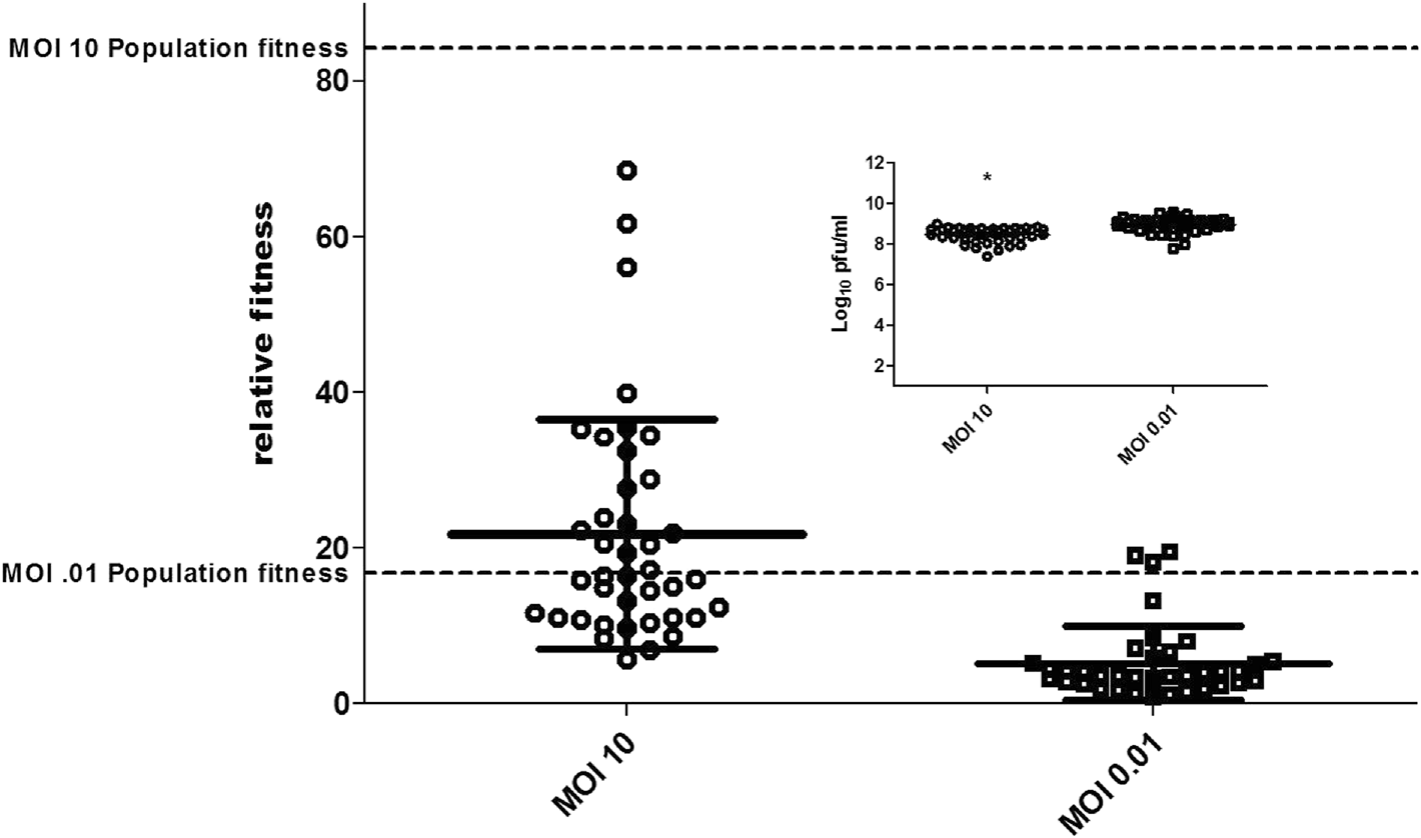
**Fitness distributions of WNV CP40 with or without co-infection on mosquito cell culture.** Individual data points represent relative fitness values of 40 clones isolated following 24 h growth at MOI 10 (with co-infection) or 0.01 (without co-infection). Solid lines represent means +/- standard deviations and dashed lines represent relative fitness values of combined populations. The inset graph depicts individual viral titers of clones following 72 h growth on mosquito cell culture. Significantly higher relative fitness values and significantly lower viral titers were measured in the MOI 10 group (t-tests, p < 0.05), as indicated by the *.

In order to equate changes in genetic diversity to phenotypic change and directly assess how levels of co-infection alter fitness landscapes, the relative fitness of 40 biologic clones was assessed for WNV CP40 following an additional passage of 24 h on mosquito cells at either an MOI of 10 or 0.01. With the starting population already characterized (Figure 2), a direct assessment of how co-infection functions to alter the phenotypic diversity in the WNV swarm could be achieved. Viral titers of individual variants following plaque isolation and 72 h growth on mosquito cell culture were found to be modestly higher for the MOI 0.01 group (Figure 5 inset; *t*-test, t = 6.0, df = 80, p < 0.0001). Competition assay results demonstrate that the level of co-infection in a single round of amplification can substantially alter the composition of variants maintained in the population (Figure 5). The mean relative fitness value for the variants derived from the MOI 10 infection was 21.8, which, despite the relatively lower individual viral titers, was significantly higher than variants derived from the MOI 0.01 infection (mean =5.2; *t*-test, t = 6.7, df = 75, p < 0.0001). In addition, more phenotypic diversity was maintained in the MOI 10 group relative to the MOI 0.01 group (standard deviations =14.7 and 4.8, respectively; F-test for variances, F = 9.57, p < 0.0001). Relative fitness values ranged from 5.6 to 68.5 in the MOI 10 group and 0.87 to 19.4 in the MOI 0.01 group. The relative fitness value of the combined population for the MOI 0.01 group was 16.9, which was higher than all but 3 (92.0%) of the variants. None of the individual variants in the MOI 10 group had relative fitness values that measured as high as the combined population fitness (84.3; Figure 5).

## Discussion

The fact that arboviruses almost exclusively possess RNA genomes suggests that the maintenance of genomic flexibility may provide a considerable advantage for viruses that require host cycling, yet a more complete understanding of the phenotypic consequences of diverse arboviral swarms is needed in order to equate RNA swarm breadth and composition to specific adaptive and evolutionary outcomes. This requires both an ability to correlate genomic diversity to intrahost fitness landscapes and an understanding of the potential for interactions among viral genomes and proteins. Here, we determined clonal fitness landscapes of a highly genetically diverse mosquito cell-adapted WNV strain and evaluated how altering the capacity for interactions among variants affects mutant swarm dynamics and swarm fitness. The somewhat counterintuitive observation of accumulating genetic diversity of WNV in the face of adaptation ([22]; Figure 1) can be explained in two ways; (i) A mutationally robust population has formed around a high fitness master sequence, or (ii) populations of variants of lower fitness values are maintained in the population via complementary interactions with more highly adapted variants. Our results demonstrate that the production and maintenance of genetic diversity in mosquito cell-adapted WNV is likely a result of a combination of these factors. Specifically, although each clone is predicted to have an average of 12 mutations relative to the consensus sequence [WNV CP40 nt diversity = 0.11%; [22]], as predicted in a robust viral population, there is substantial phenotypic redundancy, with many clones displaying similar values for relative fitness (Figure 2). Recent characterization of a mutagenized poliovirus swarm demonstrated the capacity for an RNA virus population to occupy such a neutral fitness landscape [24]. In contrast, results here also demonstrate significant fitness variation among outliers in the WNV CP40 population, with a greater than 15-fold disparity measured in relative fitness values of individual clones within the population. Similar to previous studies with VSV, the majority of variants were found to have fitness values lower than that of the combined population [25]. These data suggest that cooperative interactions among variants may function to maintain phenotypic diversity, yet also suggest that there may be a limit to the potential of complementation, as all variants possess at least modest adaptation to mosquito cells.

Previous studies suggest that arboviruses in natural systems may have the capacity to utilize complementation to maintain non-viable variants in co-infecting cells [10-12], suggesting that such interactions could also readily occur among variants with more intermediate fitness levels. Experimental studies with VSV have shown that relative fitness is MOI-dependent due to increased levels of strain complementation [13]. Our results show a similar trend, i.e. an increased capacity for the lower fitness WNV MARM to remain in the population when levels of co-infection are high (Figure 3a). Further, we demonstrate that this is not just dependent on initial MOI, but also on viral titer during a single infection (Figure 3b). Specifically, as viral replication increases, increasing co-infection effectively decreases the pace at which lower fitness mutants are selected against and, therefore, the capacity for high fitness variants to experience selective sweeps. Related studies with the bacteriophage Φ6 also demonstrate that co-infection can weaken selection, yet interactions with variants of this virus are further complicated by its capacity to readily reassort [26]. With a single open-reading frame, WNV is not capable of reassortment, nor is it thought to readily recombine [27,28]. Although the full genomes of output variants from MOI studies were not sequenced here, the partial sequences again fail to identify evidence of recombination, suggesting interactions likely occur on the level of viral proteins. Decreased strength of selection during single mosquito infections could be particularly advantageous to arboviruses as it would increase the capacity to maintain vertebrate-specific variants which might otherwise be purged. Previous studies with WNV CP40 demonstrated that this strain accrued no obvious phenotypic cost as a result of mosquito cell adaptation in vertebrate hosts *in vitro* or *in vivo*[20,29]. It remains to be seen how widespread this phenomenon is in nature, but it is possible that it could contribute to the slower than expected pace of consensus level evolution of arboviruses which is often attributed to the conflicting selective pressures resulting from host cycling [30,31].

Our results confirm that increased co-infection results in both increased genetic and phenotypic variation (Figures 4 and 5). Although one could argue that the genetic diversity differences could be attributed to variable bottleneck size, an MOI of 0.01 in these studies still requires approximately 1 million WNV particles. In addition, the differences in mean hamming distances of the MOI 10 variants, which represent an increased likelihood to retain more genetically distant haplotypes, cannot be explained by variation in bottleneck size (Figure 4). Taken together, these data demonstrate the potential for complementation to decrease short-term evolvability in one system by dampening the force of selection, but potentially increase long-term evolvability and phenotypic flexibility by maintaining high levels of diversity.

The difference in distributions of relative fitness values among individual variants with or without co-infection demonstrates the profound effect that interactions can have in shaping fitness landscapes (Figure 5). Quantification of viral titers of WNV clones isolated from these populations suggest only modest phenotypic differences, with mean viral titer ~ 0.5 log_10_ pfu/ml lower for the MOI 10 group (Figure 5). This result is consistent with the prediction that when co-infection is common, variants that have moderately inferior replicative ability are more likely to be maintained in the population as a result of complementation. Surprisingly, despite these lower viral titers, relative fitness values were substantially higher for the variants isolated following co-infection (Figure 5). This result highlights how individual replicative ability is very much distinct from fitness in competition and, therefore, how misleading using such measures as a surrogate for viral fitness can be. Here, high fitness variants do not grow to higher titers in the absence of competition, yet are exceedingly better at competing for cellular resources. The MOI 10 population clearly demonstrates variant complementation, as the population fitness in this case is greater than any of its individual components. These data are unique in that they suggest not just that individual genotypes of lower fitness can be retained by complementation, but also that there may be a population benefit as a result of cooperative interactions. These cooperative interactions, i.e. interactions that result in a net gain in swarm fitness, could result from many mechanisms ranging from a negative effect on the competitor’s fitness imposed by the group, to a ‘division of labor’ strategy existing between members of the swarm. In the latter scenario, individual genotypes could act as specialists coding for proteins that vary in their capacity to carry out different functions in the WNV life cycle. For unique variants in the absence of co-infection, it is feasible to imagine that there could be some functional trade-offs via antagonistic pleiotropy, yet if products of variants are readily shared among co-infecting particles such trade-offs could potentially be overcome. Although there are no specific examples of functional antagonistic pleiotropy for an arbovirus during infection of a single host, experimental evolution studies clearly demonstrate that there are many ways for an arbovirus to enhance fitness, and despite the pace and breadth at which these viruses can survey sequence space, consensus genotypes from highly fit strains often possess dissimilar changes in variable genomic regions [14,16,32].

To determine the relevance of cooperative interactions in natural systems there remain many questions to answer. First, does co-infection occur frequently enough in nature such that interaction could significantly contribute to alterations in arbovirus mutant swarms and, under what conditions would cooperative interactions be selected for? It has been shown that superinfection exclusion can occur with WNV in mammalian cell culture, but also that WNV can quickly evolve resistance to exclusion [33]. Little is known about the extent of co-infection in natural systems. Studies with Φ6 suggest intrahost competition could ultimately select for selfish genotypes if levels of co-infection are high, yet the constantly changing selective pressures arboviruses face in nature may not permit this [34]. Further, it is not clear if the potential costs of cooperative interactions, such as decreased strength of selection for fast replicating variants would outweigh the benefit in mosquito hosts, yet if *in vitro* results presented here are also observed *in vivo*, populations of cooperating arbovirus genotypes may have the added benefits of both higher population fitness and increased phenotypic flexibility in the face of ever changing landscapes. Studies utilizing new technology to track large populations, such as recent work with poliovirus, could provide a more comprehensive characterization of dynamic RNA arboviral swarms which could complement phenotypic characterization *in vivo*[24]. Although results presented here do not depict the full complexity of the arboviral swarm and its interactions with natural hosts, these data demonstrate the profound effects variant interactions could have on arbovirus evolution and adaptation and provide a baseline by which to study the impact of this phenomenon in natural systems.

## Conclusions

Our results demonstrate that although there is mutational robustness and phenotypic redundancy in the WNV swarm, genetic diversity also corresponds to substantial phenotypic diversity in terms of viral fitness. In addition, data demonstrate that increasing levels of co-infection can lead to widespread strain complementation which acts to maintain both genetic and phenotypic diversity and significantly dampen the strength of selection for high fitness variants. Lastly, we show that these interactions may be cooperative in that they lead to population fitness levels which exceed the fitness of any of the individual components of the swarm. Overall, these studies demonstrate the profound effects interactions can have on RNA virus evolution and adaptation, and provide a baseline by which to study the impact of this phenomenon in natural systems.

## Methods

### Virus strains and isolation of biologic clones

The biologic clone of WNV used as the parental strain for passages studies was isolated from WNV NY003356 by three rounds of plaque purification on Vero cells (ATCC #CCL-81) and WNV CP40 was obtained by 40 sequential passages on C6/36 mosquito cells (ATCC #CRL-1660) as previously described [19]. Both the biologic clone and strains derived from the initial passage on mosquito cell culture were previously found to be highly genetically homogeneous (Figure 1; [6]). The WNV monoclonal antibody resistant mutant (WNV MARM) was isolated in the presence of WNV MAb 5 H10 (BioReliance Invitrogen Bioservices #81-003) as previously described, and retained both equivalent growth kinetics and fitness relative to both the parental strain and the initial mosquito cell passage [19]. All plaque titrations were completed in duplicate on Vero cells as described elsewhere [35]. Biologic clones for phenotypic characterization were isolated at random from two 6-well plates following plaque titration of WNV strains to 10-20 pfu/well. Forty single plaques per strain were re-suspended in 100ul BA-1 and immediately inoculated onto confluent monolayers of C6/36 cells for amplification. Following 72 h of growth, cloned strains were harvested and stored at -80°C for subsequent titration and competition assays.

### Infectivity in mosquito cell culture

In order to assess the level of co-infection occurring in C6/36 cells infected with WNV CP40, a combination of quantitative RT-PCR and flow cytometry was used. C6/36 cells were infected with WNV CP40 at an MOI of 10 on 6-well plates for 12 h. WNV RNA particles were quantified before and after incubation using TaqMan qRT-PCR (Applied Biosystems). Specifically, RNA was extracted using the QIAamp viral RNA extraction kit (Qiagen) from both initial inoculum (time 0) and incubated inoculum following washing (1 h post infection). The difference in these measures was used to estimate the number of WNV particles entering the cells. Cells were then trypsinized, placed into a flow tube to the concentration of 1x10^6^, and fixed with 4% paraformaldehyde (PFA) at 4°C for 20 min. PFA was removed by centrifugation and then re-suspended in 500 ul of saponin and again incubated at 4°C for 20 min. After centrifugation to remove saponin, WNV mouse hyperimmune ascities fluid (CDC) polyclonal antibodies (1:100) was added and incubated in the dark at 4°C for 30 min. Following additional washes with saponin, goat anti-Fitc (KPL) IgG (1:50) was added and incubated for 30 min at 4°C. After 3 washes with saponin, cells were resuspended in 250 PBS +1% FBS and fixed overnight with 250ul 4% PFA. Cells were then analyzed using the Becton Dickinson Facscan (BD bioscience) and levels of WNV positive cells were quantified by reporting the proportion of cells with fluorescent signal exceeding cells derived from a negative control well inoculated with media only.

### *In vitro* competition assays

Relative fitness of WNV was evaluated by competition assays on C6/36 mosquito cells using a modification of previous studies [36]. Briefly, confluent cell monolayers in six-well plates were infected in duplicate with a 1:1 mixture of control (WNV MARM) to test virus at the desired MOI (0.01 PFU/cell unless otherwise denoted), based on Vero cell titer. WNV MARM has been shown in multiple assays to have equivalent fitness to both the unpassed WNV biological clone and the initial passage in mosquito cell culture [18]. After a 60-min absorption period at 28°C the infected monolayers were washed three times and overlaid with 3 ml of maintenance medium. Medium from the infected cultures was harvested at 24, 48, and 72 h post infection, diluted 1:10 in growth medium supplemented with 20% FBS, and frozen at -80°C for subsequent titration. The quantity of control and test virus was obtained by plaque titration in the presence (MARM control titer) or absence of Mab (total titer), and the reported relative fitness refers to the mean output ratio of test:control from duplicate assays.

### Molecular cloning and sequencing

Production and analysis of clones was performed basically as previously described [20]. RNA was extracted from infected specimens with QIAamp viral RNA extraction kit (Qiagen) and RT-PCR was conducted using primers designed to amplify the 3' 1311 nt of the WNV envelope (E) coding region and the 5' 3248 nt of the WNV non-structural protein 1 (NS1) coding region. RT was performed with Sensiscript RT (Qiagen) at 45°C for 40 min followed by heat inactivation at 95°C for 5 min. The resulting cDNA was used as a template for PCR amplification. WNV cDNA was then amplified with a ‘high-fidelity’ protocol using *Pfu*Ultra (published error rate = 4.3 X 10^-7^; Stratagene), according to the manufacturer's specifications. PCR products were visualized on a 1.5% agarose gel and DNA was recovered by using a MinElute Gel Extraction kit (Qiagen) as specified by the manufacturer. The recovered DNA was ligated into the cloning vector pCR-Blunt II-TOPO (Invitrogen) and transformed into One Shot TOP10 Electro-competent *E.coli* cells according to the manufacturer's protocol. Kanamycin resistant colonies were screened by direct PCR using primers specific for the desired insert and plasmid DNA was purified using a QIAprep Spin Miniprep kit (Qiagen) as specified by the manufacturer. Sequencing was performed at the Wadsworth Center Applied Genomics Technology Core using ABI 3700 and 3100 automated sequencers (Applied Biosystems). Seventeen to twenty-two clones were sequenced per sample.

### Sequence and data analysis

WNV sequences were compiled, edited, and aligned using the SeqMan module of the DNASTAR software package and a minimum of two-fold redundancy throughout was used for individual sequence data. All measures of sequence diversity were based on deviation from WNV CP40 (GenBank accession number JQ918659). Normalized Shannon entropy (S_n_) was calculated based on frequency of genotypes in populations as follows: Shannon entropy (S_n_) = ∑-_i_ P_i_ lnP_i_/ln N, where P_i_ = frequency of individual genotype and N = number of clones sequenced. Sn values range from 0 (completely homogeneous) to 1 (completely heterogeneous)_._ Mean hamming distance was calculated by averaging the number of base substitutions in individual clones relative to the consensus sequence. Tests for recombination including the RDP [37], GENECONV [38], and MaxChi Smith 1992 [39]methods were performed using the Recombination Detection Program v.4.13[23]. Statistical analyses were performed using both Microsoft Excel 2003 and GraphPad Prism version 4.00.

## Authors’ contributions

All authors have read and approved the final manuscript. ATC conceived and coordinated the experiments, analyzed and interpreted the data, carried out the experiments, and wrote the manuscript. DJE, GAVS, and GGW analyzed data and carried out the experiments. LDK conceived and coordinated the experiments.
